# Musical practice as an enhancer of cognitive function in healthy aging - A systematic review and meta-analysis

**DOI:** 10.1371/journal.pone.0207957

**Published:** 2018-11-27

**Authors:** Rafael Román-Caballero, Marisa Arnedo, Mónica Triviño, Juan Lupiáñez

**Affiliations:** 1 Mind, Brain and Behavior Research Center (CIMCYC), University of Granada, Granada, Spain; 2 Department of Psychobiology, University of Granada, Granada, Spain; 3 San Rafael University Hospital, Granada, Spain; 4 Department of Experimental Psychology, University of Granada, Granada, Spain; Anadolu University, TURKEY

## Abstract

Aging is accompanied by cognitive decline, although recent research indicates that the rate of decline depends on multiple lifestyle factors. One of such factors is musical practice, an activity that involves several sensory and motor systems and a wide range of high-level cognitive processes. This paper describes the first systematic review and meta-analysis, to our knowledge, of the impact of musical practice on healthy neurocognitive aging. The inclusion criteria for the review required that studies were empirical works in English or Spanish that they explored the effects of musical practice on older people; they included an assessment of cognitive functions and/or an assessment of brain status; and they included a sample of participants aged 59 years or older with no cognitive impairment or brain damage. This review led to the selection of 13 studies: 9 correlational studies involving older musicians and non-musicians and 4 experimental studies involving short-term musical training programs. The results of the meta-analysis showed cognitive and cerebral benefits of musical practice, both in domain-specific functions (auditory perception) and in other rather domain-general functions. Moreover, these benefits seem to protect cognitive domains that usually decline with aging and boost other domains that do not decline with aging. The origin of these benefits may reside, simultaneously, in the specific training of many of these cognitive functions during musical practice (*specific training mechanism*), in the improvement of compensatory cognitive processes (*specific compensatory mechanism*), and in the preservation of general functions with a global influence on others, such as perceptual capacity, processing speed, inhibition and attention (*general compensatory mechanism*). Therefore, musical practice seems to be a promising tool to reduce the impact of cognitive problems associated to aging.

## Introduction

The world’s population is currently experiencing a progressive aging process [1]. This demographic change poses new challenges for today’s societies, which must address the difficulties of aging (such as age-related neurocognitive decline) and identify the factors that can offer protection against these problems.

The existence of age-related cognitive and brain decline, which become more marked around the age of 60 [2–4], is well established today. Over the years, many cognitive functions tend to decline, such as processing speed, inhibition, attention, working and episodic memory, semantic fluency, visuospatial and visuoconstructional abilities, and executive functions [2,4–7]. Most of them are part of the construct known as ‘fluid cognition’ and are considered to experience a progressive decline throughout life beginning in early adulthood [4,7]. By contrast, skills based on the accumulation of knowledge through experience (i.e., ‘crystallized cognition’), such as vocabulary or general information, tend to be maintained or even improve with age [4,7].

According to certain models of aging, most age-related differences in cognitive measures are associated with changes in a small group of functions. Specifically, there is evidence supporting the role of sensory capacity [8], processing speed [9] and inhibition [10] as possible mediators of the effects of aging on many other cognitive processes. A decline in sensory capacity seems to affect the early stages of processing, while slower processing seems to lead to incomplete later operations and reduce the amount of information available simultaneously [9]. In fact, processing speed acts as a mediator of many age-sensitive functions [11,12], including functions that involve both fluid and crystallized abilities, such as phonological fluency [13] and naming [14]. Moreover, inhibitory mechanisms seem to be central to the efficiency of working memory, limiting the entry of irrelevant information or rejecting irrelevant information that has gained access [10]. Therefore, inhibition could be important for avoiding distractions and also for speech comprehension, memory and flexibility. Finally, the increased complexity of the tasks used to assess all these functions has been found to affect the performance of older individuals more than that of younger ones (i.e., *complexity effect*) [11]. This effect seems to be mainly due to the decline in high-level processes (e.g., working memory).

In parallel, brain aging involves changes such as the loss of gray and white matter volume [15–18], as well as declines in white matter integrity [19–21]. At the microstructural level, rather than loss of neurons, which is estimated to be low [22], cell shrinkage, loss or regression of dendritic arborization and dendritic spines, and demyelination seem to occur (for a review, see [23]).

However, there is high heterogeneity in cognitive trajectories, as some individuals exhibit cognitive decline while others remain cognitively healthy across older age [24,25]. It appears that age-related cognitive decline is neither inevitable nor irreversible. Some of these differences may be due to protective genetic factors [26]. The concept of ‘cognitive reserve’ has also been proposed to explain the frequent discrepancy between an individual’s degree of brain pathology (or age-related natural decline) and the functional and cognitive deficits observed [27]. Thus, engaging in certain stimulating activities throughout life helps to reduce the impact of brain diseases and cognitive aging. Education, physical exercise, occupation and engagement in intellectually stimulating leisure activities have all been associated with a reduced risk of dementia [28], as well as with neurocognitive benefits [29–32]. Since there is evidence that neurogenesis [33] and plasticity processes [34] still occur in the brain of older adults, it appears that these lifestyle factors may continue to produce benefits during the aging process.

Musical practice, that is, musical training and performance, is one of the activities that is considered to contribute to cognitive reserve. Playing an instrument involves multiple sensory and motor systems and requires a wide variety of higher-level cognitive processes [35]. Musical practice not only involves high sensorimotor integration; it also seems to be an optimal cognitive activity since it involves regular and motivated practice of progressive difficulty, with constantly renewed stimuli and tasks that represent continuous challenges for the individual [36,37]. Accordingly, lifelong musical practice has been associated with a lower risk of dementia and mild cognitive impairment [28], even when the contribution of genetics is controlled for [38]. Moreover, long-term musical practice has also been associated with multiple cognitive advantages in adult musicians. Some of these advantages occur in functions that could be considered specific to skills improved through musical performance (i.e., domain-specific functions), such as a more robust and efficient auditory processing of musical and speech stimuli [39–44]. However, many of the benefits observed extend to more general cognitive functions (i.e., domain-general functions), such as processing speed [45], inhibition [45,46], attention [44,47], episodic memory [48], working memory [48], visuospatial ability [49,50] and language [51,52].

This is accompanied by brain changes, as shown by the increase in gray matter volume in perceptual, somatosensory and motor-related regions, as well as in high-level functions areas [53,54]. Additionally, musicians also exhibit benefits in the white matter, such as in the corpus callosum and the arcuate fasciculus, among others [55,56].

Thus, musical practice may be a potential tool for mitigating both the impact of age-related non-pathological cognitive changes as well as the incidence of dementias. However, to our knowledge, no systematic reviews have been conducted so far in the field of aging. The aim of this research was to conduct a systematic review and meta-analysis in order to compile the most relevant data to date and draw the first conclusions on the impact of musical practice on cognitive and cerebral aging.

To this end, we decided to include studies with both an experimental and correlational design, given their complementarity: experimental methodology involves randomization and thus allows causal relationships to be established; by contrast, correlational designs offer the possibility of analyzing samples with a higher and more extensive level of musical practice over longer periods of time. Unfortunately, few experimental studies exist on this subject. However, these designs provide crucial evidence to clarify issues that are not clearly explained by correlational designs (i.e., whether cognitive improvements are due to practice or, conversely, a better cognitive status leads to a greater involvement in this activity). In addition, experimental studies are also interesting in themselves, as they can elucidate whether a late onset of the activity still provides neurocognitive benefits.

## Methods

### Literature search

**Fig 1:**
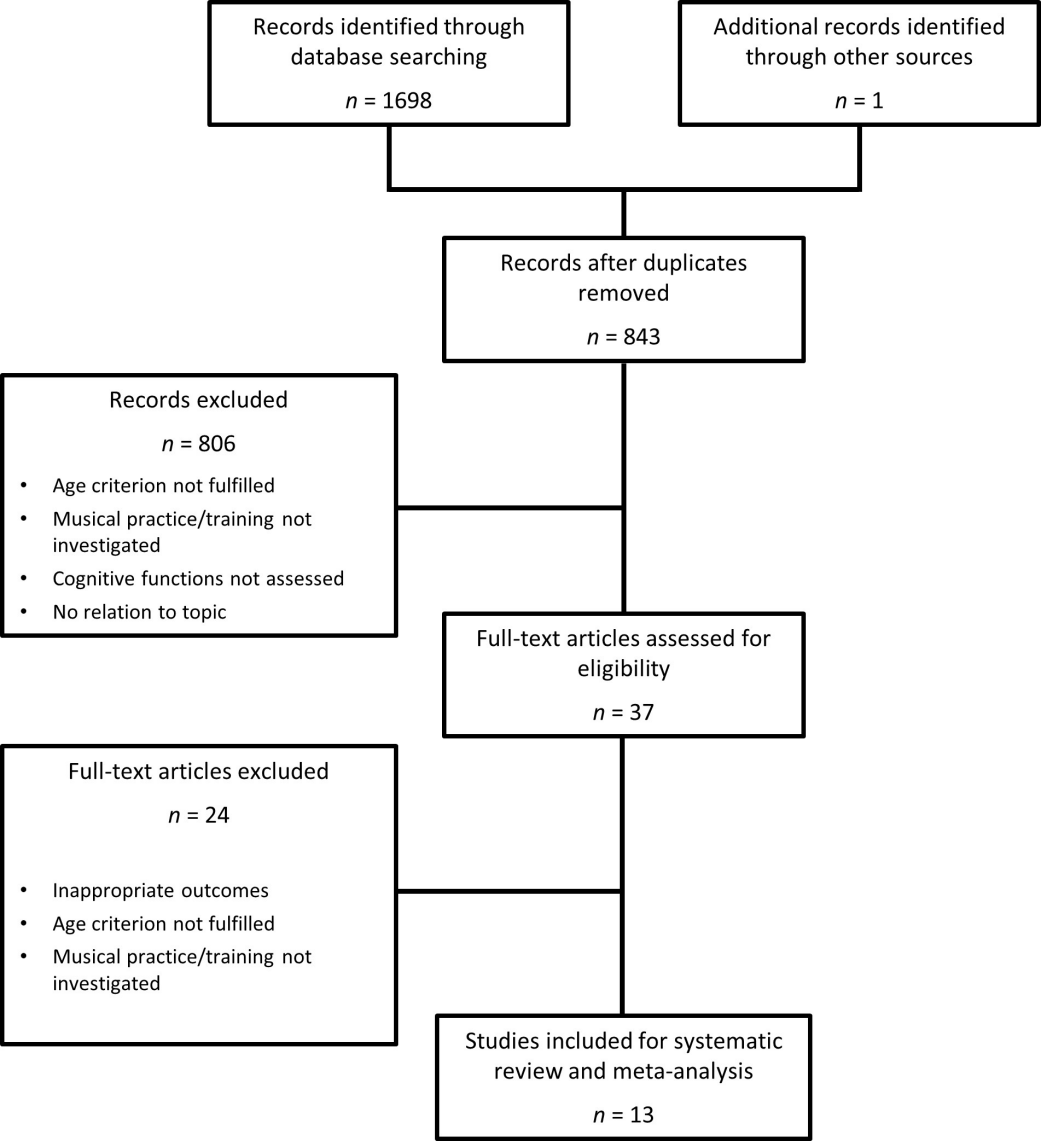
PRISMA flowchart of the studies included in the systematic review and meta-analysis.

A systematic review was conducted following the recommendations of PRISMA (see S1 Table and [57]). As a first step, we consulted the Ovid, ProQuest, PubMed, Scopus and Web of Science databases. The search equation used was (aging OR older* OR elder*) AND (music* OR musical practice OR musical training) AND (cogniti* OR cognitive reserve OR plasticit*). Second, we consulted references from studies on this subject. Finally, we accessed the ‘gray literature’ via Google Scholar and TESEO. The latest search was carried out in August 2018, without any time restrictions. In total, 1699 potentially interesting results were found. After removing 855 duplicates, titles and abstracts of 843 studies were screened by RRC (Rafael Román-Caballero) to exclude articles that did not meet the inclusion criteria. The full text of 37 of the selected articles was assessed for eligibility by two independent reviewers (RRC and JL, Juan Lupiáñez). Any disagreements were resolved by discussion and consensus between both researchers. Finally, 13 of these articles were selected for inclusion in the review– 4 of them in the experimental study group and 9 in the correlational study group. A PRISMA flowchart summarizing the literature search process is depicted in Fig 1 (see S1 File for the specific search procedures of each database).

### Selection criteria

The studies selected in the review met the following criteria: (1) be empirical; (2) explore the effects of musical practice on the sample; (3) include an assessment of cognitive functions and/or an assessment of brain status with a physiological recording and/or neuroimaging technique; (4) include a sample of participants aged 59 years or older with (5) no cognitive impairment or brain damage; and (6) be written in English or Spanish. The age criterion was selected on the basis of the existing evidence that cognitive [4] and cerebral [3] aging occurs most markedly around 60 years of age. We decided to extend a priori this age limit to 59 years in order to cover studies that by convention begin their range at this age.

### Data extraction and quality assessment

Coding sheets were created for recording the variables (see S2 File). By using them, basic information, design, sample, outcomes and results were obtained from each of the selected studies. In the correlational studies, the variables related to the musical experience of the sample were also extracted. In the experimental studies, information of musical training programs and the follow-up assessments were collected. Moreover, information regarding the control variables was also collected in both types of studies.

**Fig 2:**
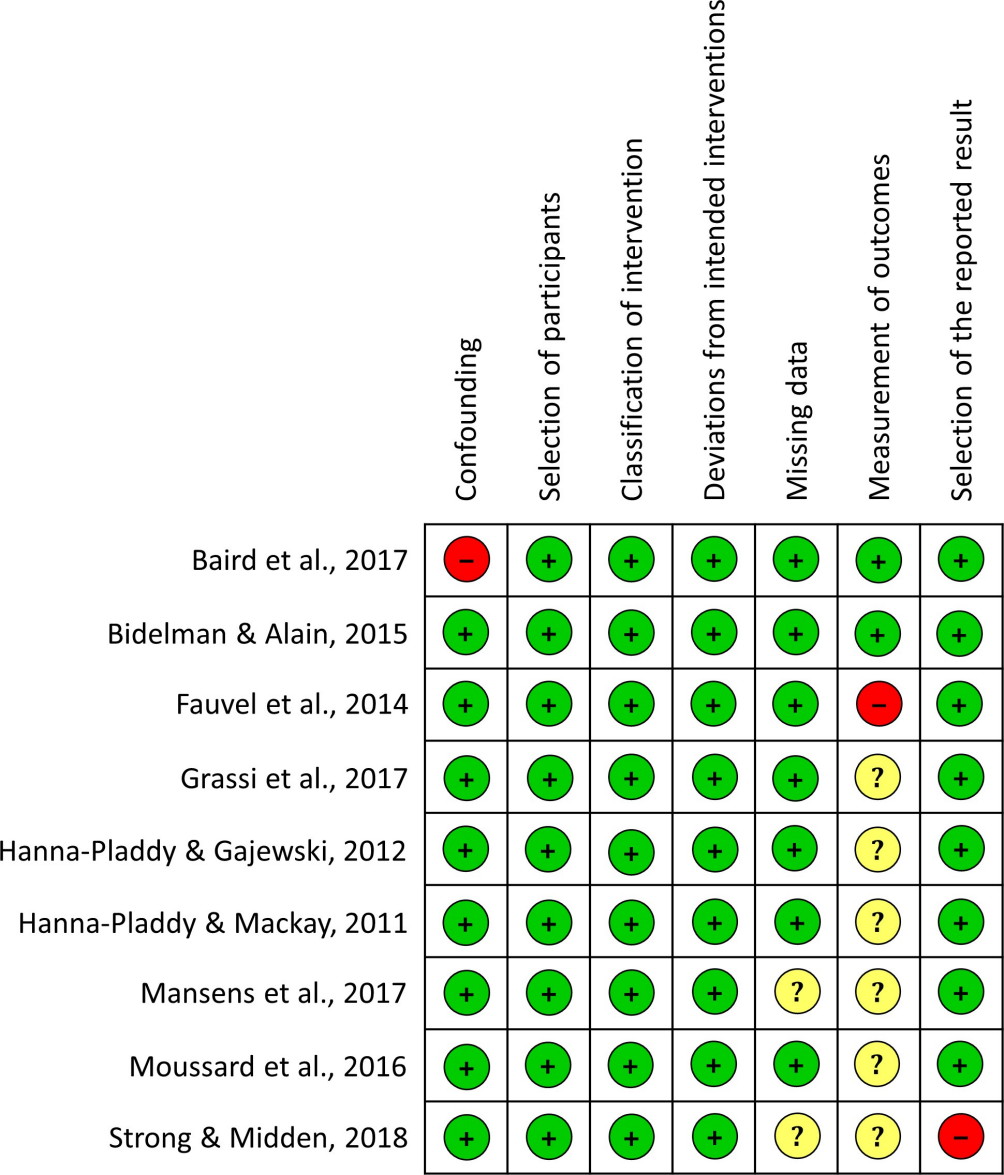
Risk of bias summary for correlational studies.

**Fig 3:**
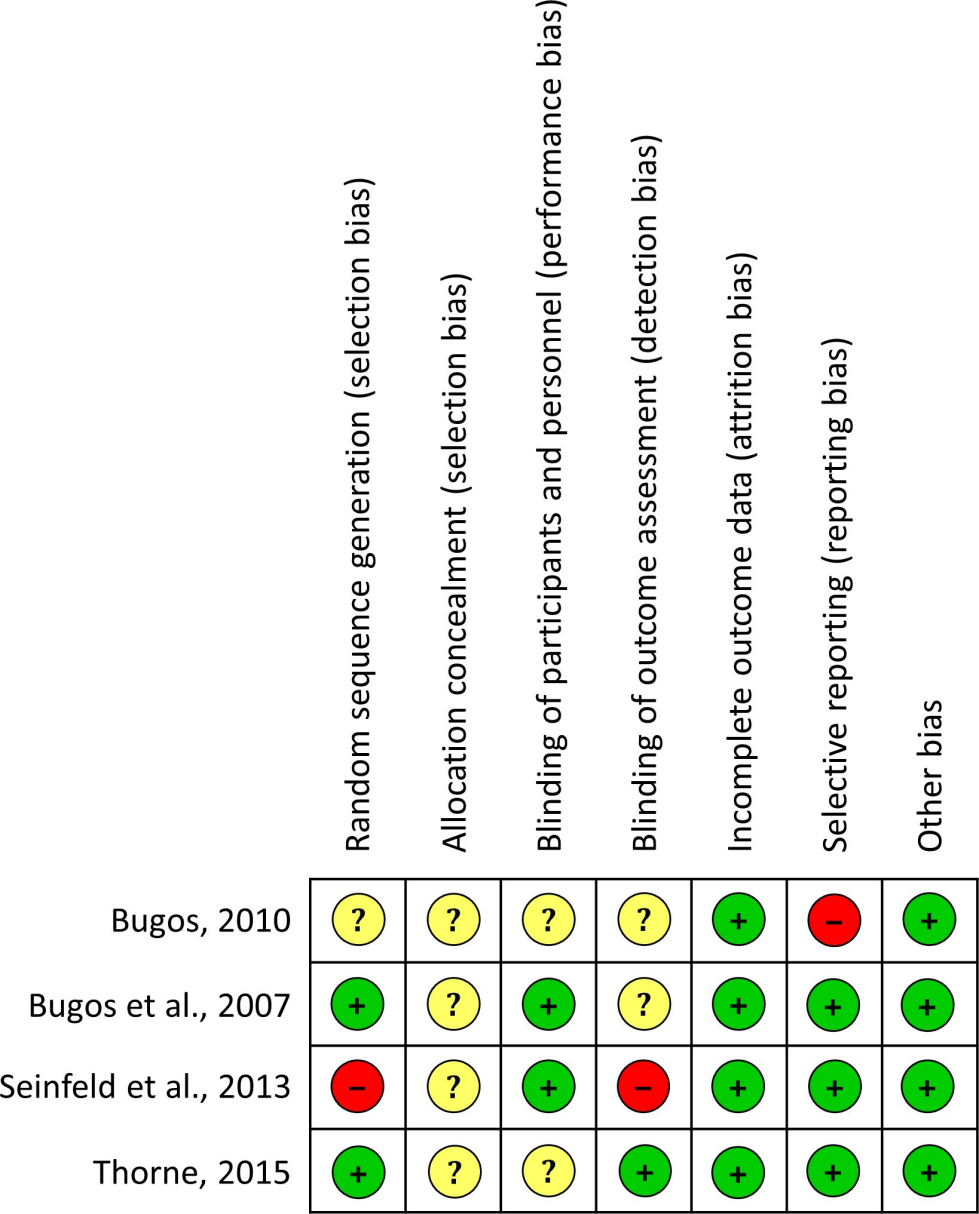
Risk of bias summary for experimental studies.

Quality assessment was also performed using the Cochrane Risk of Bias (RoB) for experimental studies [58] and the Risk of Bias in Non-randomized Studies of Interventions (ROBINS-I) for correlational studies [59]. The judgment in the different domains for each study are summarized in Figs 2 and 3.

### Statistical analysis

#### Effect size

To explore the effects of musical practice on the various cognitive functions, separate meta-analyses were performed for each function in each of the two types of studies (i.e., experimental and correlational). To this end, we used Hedge’s *g* [60] as an estimator of effect size. The *g* values were interpreted according to Cohen’s criteria: values of 0.2–0.5 are interpreted as small effect, 0.5–0.8 as medium effect, and > 0.8 as large effect [61]. In our study, a positive effect size denoted an improvement in favor of musical practice. In the experimental studies, which included both pre- and post-treatment measures, effect size was calculated following the proposal made by Morris [62]: *d* = ((M_post,T_−M_pre,T_)–(M_post,C_−M_pre,C_)) / SD_pre_, where M_post,T_ and M_pre,T_ are the post- and pre- mean scores for the treatment group, whereas M_post,C_ and M_pre,C_ are the scores for the control group, and SD_pre_ is the pooled standard deviation for the pre-test scores of both groups. In studies that included two post-treatment measures (of which one was a follow-up measure), we calculated effect size in the first post-intervention assessment. In correlational studies with more than one group of musicians, we used the scores of the group of practice with the greatest expected effect (either high activity musicians [63] or active musicians [64]).

#### Outcome measures and aggregates

As most of the studies included multiple neuropsychological measures of the same function, we decided to generate aggregates following the recommendations of Borenstein et al. [65] (see S2 Table). The aggregates were produced with the Agg function of the MAd package in R [66]. In the absence of correlations between the different measures, the default correlation of 0.5 was selected, based on Wampold et al. [67]. The metafor package for R [68] was used to conduct the univariate meta-analyses of the various cognitive functions. Given the great variability of neuropsychological outcomes and other differences between studies (e.g., age, education, musical variables), a random effects model was used for the meta-analyses. The studies used different tests involving different scales, so the standardized mean difference (SMD) and the 95% confidence interval (CI) were used as the summary measure of effect. Significance was defined as the two-sided p-value of <.05. Indeed, the results found with the multivariate method were very similar to those obtained with the chosen univariate method on aggregates.

#### Heterogeneity

To ensure a sufficient consistency between studies and the generalization of the summarized findings, the usual heterogeneity indexes were computed: Q and its chi-squared significance test, τ^2^ and I^2^. I^2^ values of 0–25% are interpreted as null, 25–50% as low, 50–75% as moderate, and <75% as high heterogeneity [65]. Due to the low number of studies included (<10 in both cases [69]), we considered it inappropriate to use funnel plots and tests such as Egger’s regression test [70] to assess publication bias.

## Results

### Correlational studies: Cognitive benefits associated with musical practice throughout life

#### Characteristics of the studies

Our research included 9 correlational studies, with a total sample of 1,530 subjects. The main characteristics of these studies are summarized in Table 1. The proportion of men and women was similar in most studies. The presence of neurological or psychiatric disorders and cognitive impairment in the studies was controlled for, and efforts had been made to generate homogeneous groups regarding age, education, IQ, and in certain cases also regarding physical activity [64,71], income [71] and social activity [64]. However, differences between the studies can be observed in age (mean age range: 67 to 74.87 years), level of education (range: 9.4 to 18.3 years of education on average), and in the variables related to musical practice (Table 2).

**Table 1:** Main characteristics of the correlational studies included in the systematic review and meta-analysis.

			Total sample			Groups	Results	
Authors	Year		Size	Age	Sex	Characteristics	Main	Others
Baird et al. [72]	2017		N = 22	≥ 65 years	–	Musicians (n = 15) Non-musicians (n = 7)	ns	–
Bidelman & Alain [73]	2015		N = 20	> 60 years	50% men	Musicians (n = 10) Non-musicians (n = 10)	–	Faster classification of speech sounds
								Brain coding of speech more efficient and robust; also possible improvement in attention
Fauvel et al. [74]	2014	Study 1	N = 68	≤ 55 years vs. ≥ 60 years	44% men	Older musicians (n = 15) Older non-musicians (n = 20) Middle-aged musicians (n = 19) Middle-aged non-musicians (n = 14)	↑ DSF ↑ D2 ↑ Letter fluency ↑ Semantic fluency	With aging: - Less decline in D2 - No decline in DSF and semantic fluency - Better letter fluency
		Study 2	N = 47	≥ 60 years	47% men	Older early musicians (n = 15) Older late musicians (n = 12) Older non-musicians (n = 20)	–	- Older musicians with early onset showed better letter fluency - Older musicians showed better semantic fluency
Grassi et al. [37]	2017		N = 40	≥ 65 years	70% men	Musicians (n = 20) Non-musicians (n = 20)	↑ VPTA ↑ LST ↑ sEFT ↑ sMRT	Better central auditory processing
								Frequency discrimination, gap detection, VPTA, LST and sMRT are good to excellent classifiers for musicians
Hanna-Pladdy & Gajewski [75]	2012		N = 70	≥ 59 years	–	Musicians (n = 33) Non-musicians (n = 37)	↑ LNS ↑ Letter fluency D-KEFS ↑ CVLT-II SDFR ↑ JLO ↑ Tower task D-KEFS (rule violations) Trend in GP	Trends in ROCF delayed recall and D-KEFS letter fluency in currently active musicians (they also have more years of practice)
								In partition analyses, predictors:
								JLO - Education ≥ 17 years - In education < 17 years, current practice
								LNS Age of onset < 9 years
Hanna-Pladdy & MacKay [63]	2011		N = 70	≥ 60 years	40–50% men	High activity musicians (n = 22) Low activity musicians (n = 27) Non-musicians (n = 21)	↑ VRII ↑ TMT-A ↑ TMT-B ↑ BNT Trends in SS and letter fluency	Cognitive performance (VRII, TMT-B and BNT) correctly classifies 57.1% of participants (77.3% of high activity musicians) into the 3 groups.
								Age of onset is the best predictor of VRI, years of practice of VRII (followed by age of onset), age of TMT-A (followed by years of practice) and TMT-B (followed by current practice), and type of training of BNT
Mansens et al. [71]	2017		N = 1101	≥ 64 years	52% men	Musicians (n = 277) Non-musicians (n = 824)	↑ Alphabet coding Task-15 ↑ Letter fluency ↑ DSF ↑ DSB ↑ AVLT-Learning Trend in AVLT-Delayed recall	Playing an instrument: - > singing and non-musicians in Alphabet coding Task-15 - > non-musicians in DSB - > non-musicians in AVLT-Learning
Moussard et al. [76]	2016		N = 34	≥ 59 years	47% men	Musicians (n = 17) Non-musicians (n = 17)	↑ Go/No-go (Errors)	Overall, more inhibitory ability and more anterior activation:
Strong & Midden [64]	2018		N = 58	≥ 65 years	53% men	Active musicians (n = 32) Former musicians (n = 12) Non-musicians (n = 14)	↑ Stroop-1 D-KEFS ↑ Stroop-3 D-KEFS ↑ Stroop-4 D-KEFS ↑ BNT ↑ Letter fluency	–

**DSF**: Digit Span Forward; **VPTA**: Visual Pattern Test Active; **LST**: Listening Span Test; **sEFT**: short Embedded Figures Test; **sMRT**: short Mental Rotation Test; **LNS**: Letter-Number Sequencing; **D-KEFS**: Delis-Kaplan Executive Function System; **CVLT-II SDFR**: California Verbal Learning Test-II Short Delay Free Recall; **JLO**: Judgment of Line Orientation; **ROCF**: Rey Osterrieth Complex Figure; **GP**: Grooved Pegboard; **VRI &VRII**: Visual Reproduction I & II; **TMT**: Trail Making Test; **BNT**: Boston Naming Test; **SS**: Spatial Span; **DSB**: Digit Span Backward; **AVLT**: Auditory Verbal Learning Test.

**Table 2:** Variables related to the musical practice of participants in the studies included in the systematic review and meta-analysis.

Study	Professional musicians	Groups	Age of onset	Years of training	Years of practice	Current practice (%)	Current practice (h/week)
Baird et al., 2017	Yes	–	–	–	51 (22)	100%	–
Bidelman & Alain, 2015	No	–	10.8 (2.5)	11.4 (5.8)	–	100%	–
Fauvel et al., 2014; Study 1	No	Older musicians	11.2 (4.5)	–	Overall:	100%	9.2 (6.6)
		Middle-aged musicians	8.4 (3.7)	–	38.12 (17.7)	100%	15.3 (12.6)
Fauvel et al., 2014; Study 2	No	Older musicians with early onset	11.2 (4.5)	–	–	100%	9.2 (6.6)
		Older musicians with late onset	42.7 (11)	–	25.8 (12.3)	100%	8.1 (7.2)
Grassi et al., 2017	Yes	–	–	–	60.3 (9.96)	100%	–
Hanna-Pladdy & Gajewski, 2012	No	–	9.3	4	37	51.5%	–
Hanna-Pladdy & MacKay, 2011	No	High activity musicians	9.7 (7.2)	3.5 (0.96)	35.5 (24.7)	45.5%	–
		Low activity musicians	10.4 (5.9)	3.3 (0.95)	3.8 (2.7)	11.1%	–
Mansens et al., 2017	–	–	–	–	–	–	–
Moussard et al., 2016	Professionals and amateurs	–	8.8 (3.8)	27.8 (19.5)	57.2 (8.4)	100%	11 (6.3)
Strong & Midden, 2018	–	Active musicians	8.4 (3.4)	9.5 (6.4)	–	100%	7.9 (6.8)
		Former musicians	8.6 (2.2)	7.9 (5.3)	–	–	–

#### Meta-analyses of specific cognitive functions

**Fig 4:**
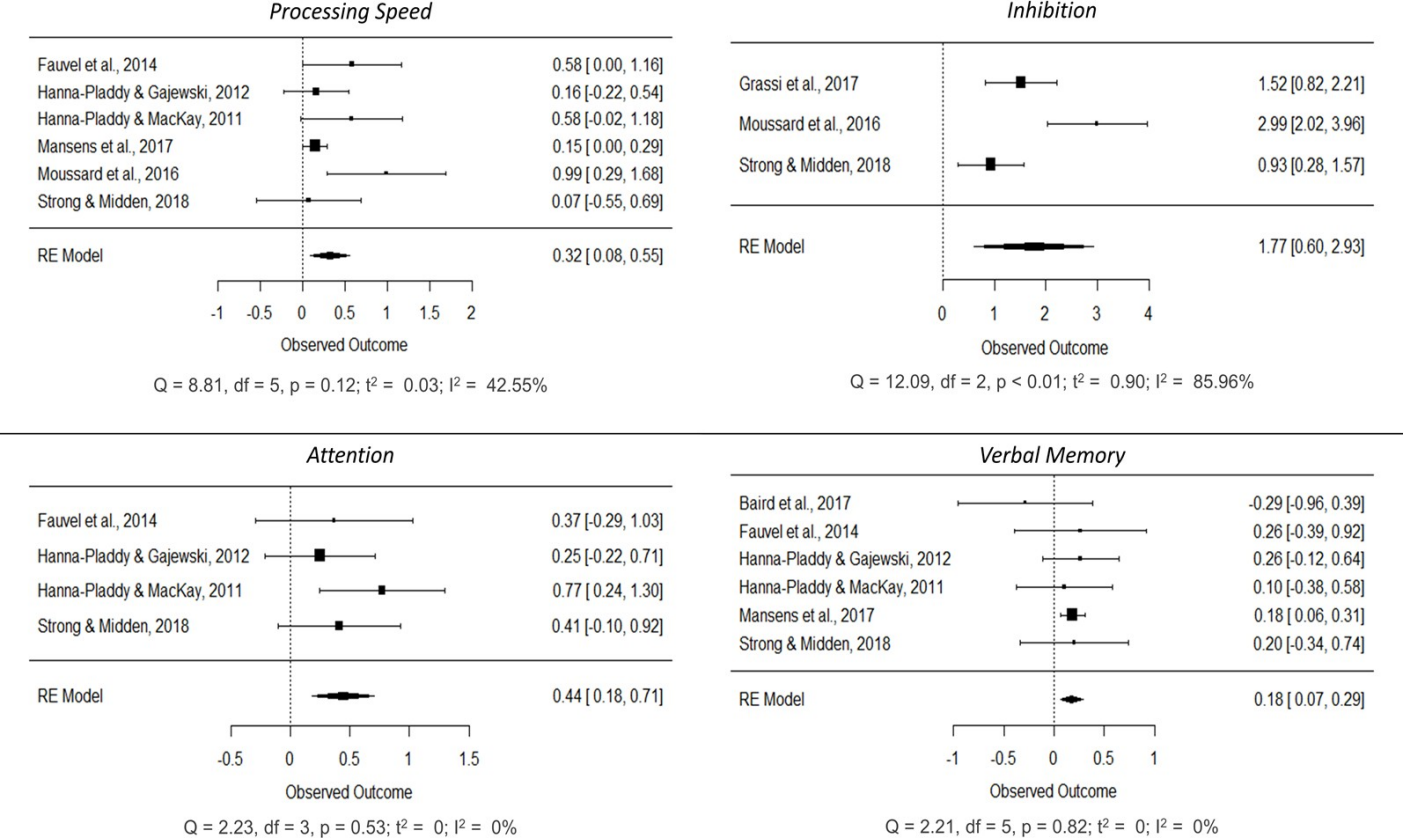
Forest plots showing cognitive improvements in processing speed, attention, inhibition and verbal memory in older adults associated with long-term musical practice.

**Fig 5:**
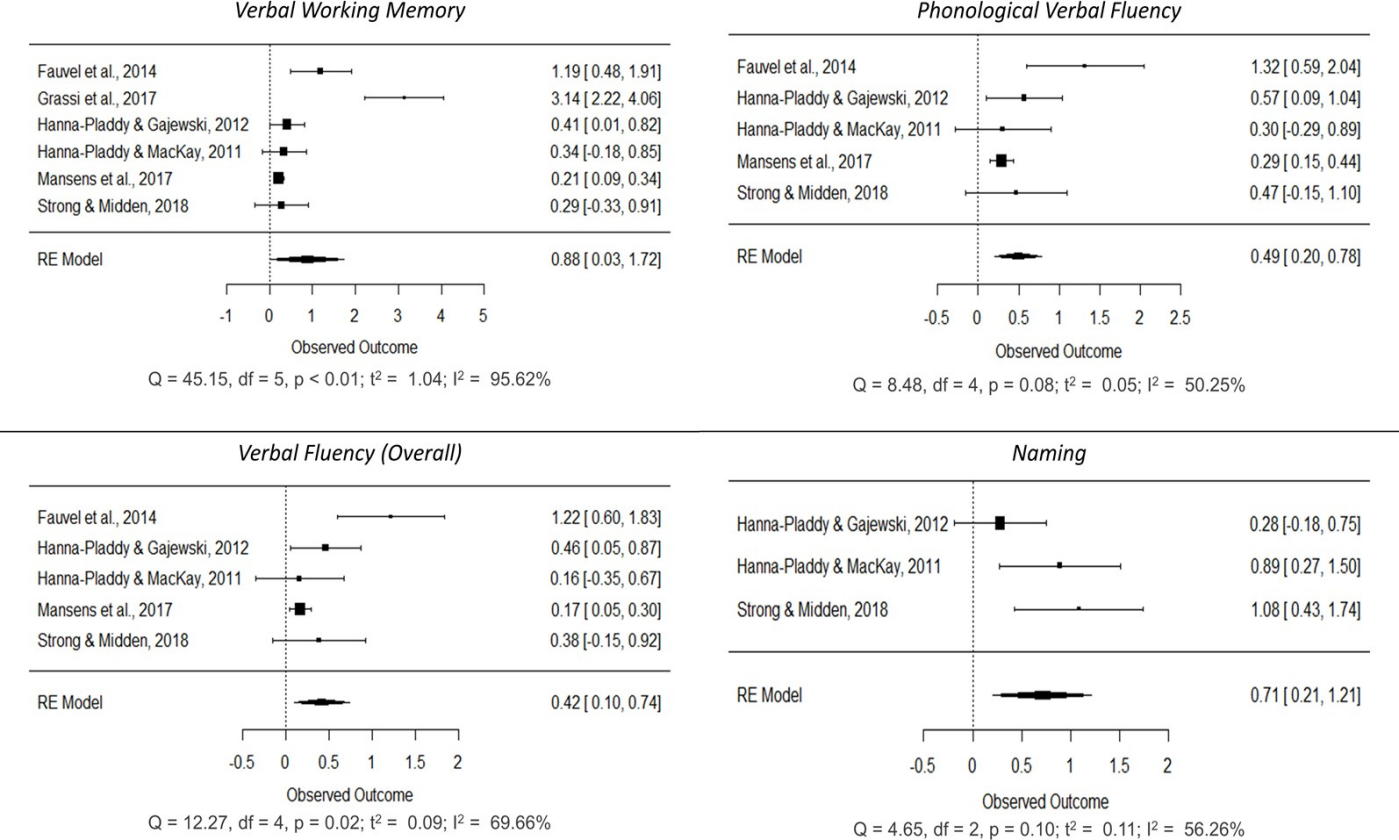
Forest plots showing cognitive improvements in verbal working memory, verbal fluency (overall), phonological verbal fluency and naming in older adults associated with long-term musical practice.

**Fig 6:**
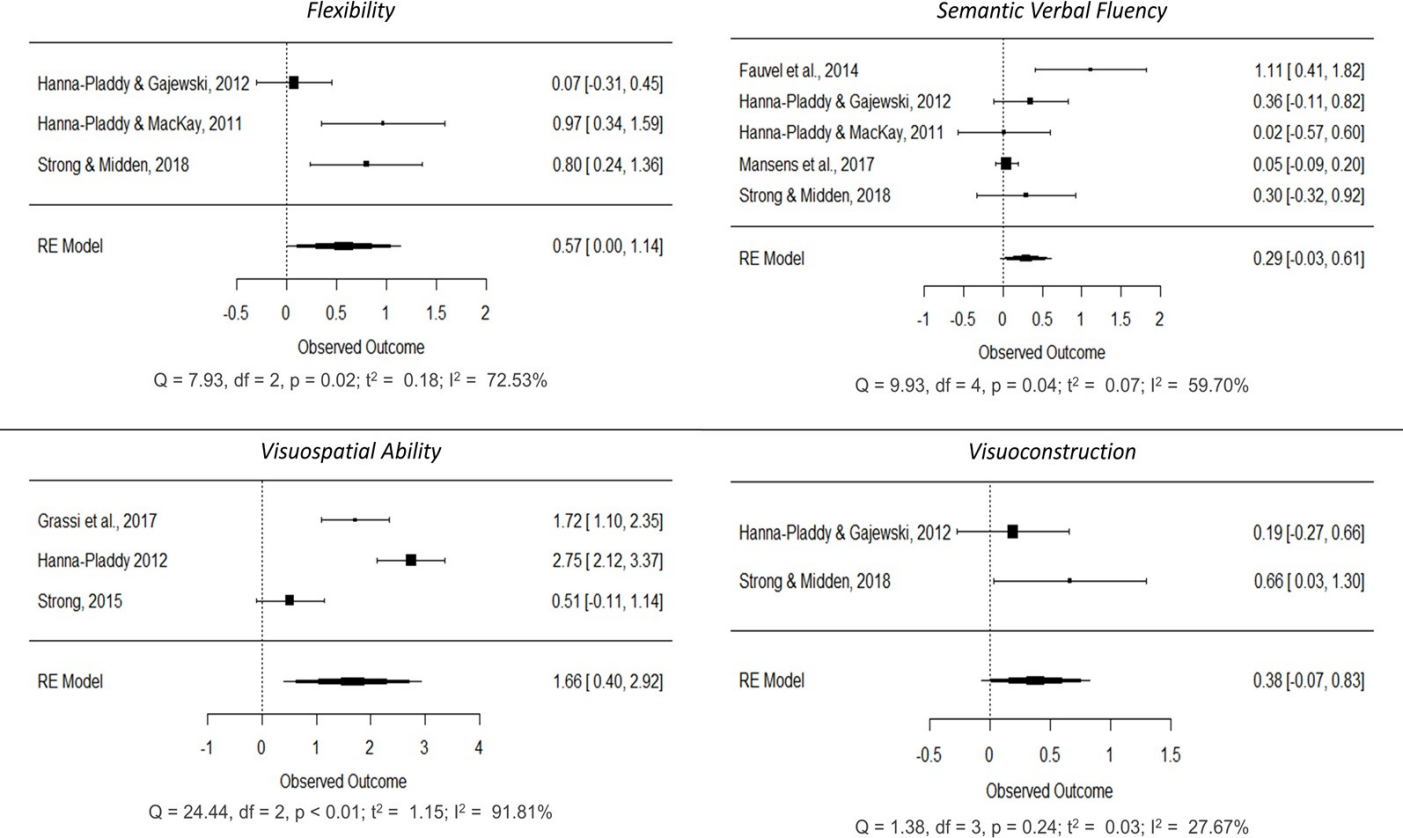
Forest plots showing cognitive improvements in flexibility, visuospatial ability, semantic verbal fluency and visuoconstruction in older adults associated with long-term musical practice.

Separate meta-analyses were conducted on each of the cognitive functions in order to explore the cognitive differences associated with musical practice. Eventually, we decided to explore basic domain-general functions (processing speed, inhibition and attention) and complex domain-general functions (verbal and visual working memory, naming, verbal fluency, verbal and visual memory, reasoning, flexibility, visuospatial ability and visuoconstruction) (Figs 4–6; forest plots of non-significant cognitive functions are reported in the S3 File). These cognitive functions were not assessed in all the studies; consequently, the total number of samples for each of the meta-analyses ranged between 116 and 1,386 participants (mean = 648, SD = 604.040). Generally speaking, positive mean effect sizes were observed in all functions, although not all of them reached significance. A general improvement was observed in all basic domain-general functions: processing speed (SMD = 0.316, 95% CI [0.082, 0.551], p < 0.01; small effect), attention (SMD = 0.441, 95% CI [0.177, 0.706], p < 0.01; small) and inhibition (SMD = 1.766, 95% CI [0.601, 2.930], p < 0.01; large). Additional improvements were observed in complex domain-general functions such as verbal memory (SMD = 0.180, 95% CI [0.070, 0.289], p < 0.01; small), verbal working memory (SMD = 0.876, 95% CI [0.027, 1.725], p = 0.043; large), overall verbal fluency (SMD = 0.418, 95% CI [0.096, 0.741], p = 0.011; small), phonological verbal fluency (SMD = 0.493, 95% CI [0.204, 0.783], p < 0.01; small), naming (SMD = 0.708, 95% CI [0.206, 1.210], p < 0.01; medium), flexibility (SMD = 0.571, 95% CI [0.003, 1.138], p = 0.049; medium) and visuospatial ability (SMD = 1.660, 95% CI [0.397, 2.924], p = 0.01; large). A positive trend was also observed in semantic verbal fluency (SMD = 0.288, 95% CI [-0.033, 0.609], p = 0.10; small) and visuoconstruction (SMD = 0.378, 95% CI [-0.072, 0.829], p = 0.079; small).

Regarding heterogeneity, only processing speed, attention, verbal memory and visuoconstruction showed a low level (I^2^ < 50%), whereas verbal fluency measures, naming and flexibility showed a significant medium effect (75% > I^2^ > 50%), and inhibition, verbal working memory and visuospatial ability exhibited a high level (I^2^ > 75%).

#### Other findings of the systematic review

The review of the included articles provided additional evidence of improvements associated with musical practice in auditory skills (i.e., frequency discrimination, duration, gaps and amplitude modulations [37]) and speech perception (i.e., faster speech sound classification and EEG-based evidence suggesting more efficient and robust coding of speech [73]). However, the advantages in perceptual processing were not observed in Moussard et al. [76] with a visual task (no changes were observed in the early components P1 and N170). This suggests that these advantages are more restricted to the auditory modality at early stages of processing. Moreover, Bidelman and Alain [73] pointed out that increases in P3 amplitude may also indicate possible attentional improvements, implying that the advantages of musicians may extend beyond pre-attentive levels.

Additionally, some EEG findings [76] also suggested a greater inhibitory capacity in musicians and a more frontal topography of the effect observed in P3, which was partly interpreted as the development of successful compensatory mechanisms in older musicians. In this regard, Fauvel et al. [74] found that cognitive benefits exhibited a different pattern depending on the specific cognitive function concerned and its sensitivity to age. In fact, the performance of musicians also declined in some tasks (d2 test), although to a lesser extent. By contrast, no decline was observed in other tasks (Digit Span Forward and semantic fluency). Finally, in this cross-sectional study phonological fluency did not show a substantial decline; it even improved with age in musicians. These results indicate that the effects of musical practice may have either a protective or enhancing effect depending on the cognitive function concerned.

The studies included also provided evidence of the relationship between variables associated with musical practice and cognitive improvements. Functions such as phonological fluency [74], visual memory [63] and verbal working memory [75] were associated with the age of onset of the activity, showing better performance with earlier age of onset. The same applied to the amplitude of P3 in no-go trials, which was linked to a higher inhibitory capacity [76]. These relationships suggest the existence of a possible sensitive period during which musical practice is likely to have stronger and more permanent effects. In this regard, Hanna-Pladdy and Gajewski [75] found that an age of onset of less than 9 years predicted a better performance in verbal working memory.

Other variables of musical practice such as the intensity of the activity [63], maintaining the practice during old age [63,75,76], type of training [63] and its duration [76] were also associated with a wide range of cognitive improvements. Finally, older adults who played a musical instrument generally showed greater advantages than singers, especially in processing speed [71].

### Experimental studies: Cognitive effects of late-onset and short-term musical practice

#### Characteristics of the studies

Four experimental studies were included (one of them was a Ph.D. dissertation [77]), forming a total sample of 126 subjects. The main characteristics of these studies are summarized in Table 3. They all covered an age range between 60 and 85 years and showed a similar proportion of men and women across the studies, although with a higher representation of women (between 65% and 78%). As in the correlational studies, the absence of neurological disorders and cognitive impairment was controlled for, as well as other influential variables (e.g., depression, psychoactive treatment and drug abuse). Although the aim was to have homogeneous groups within each study (e.g., age, education, IQ), there were differences between them in the level of education (mean years of education ranged from 13.38 to 18.1 years).

**Table 3:** Main characteristics of the experimental studies included in the systematic review and meta-analysis.

		Total sample			Groups					Results	
Authors	Year	Size	Age	Sex	Characteristics	Random assignment	Training program	Control group	Times of measurement	Main	Others
Bugos et al. [36]	2007	N = 31	60–85 years	24% men	Experimental (n = 16): piano training Control (n = 15): without treatment	Yes	6-month piano training. ½ h of individual session and 3 h of autonomous practice per week	No training, only assessment	Pre-test Post-test (6 months later) Follow-up (3 months later)	DSy TMT-B Trend in TMT difference score and DSF	–
Bugos [79]	2010	N = 46	60–85 years	22% men	Experimental (n = 24): piano training Active control (n = 22): musical listening	–	16-week piano training. 45 min. of group session and 15 min. of social activities per week. ½ h of daily practice.	16-week musical listening. 45 min. of group session and 15 min. of social activities per week. ½ h of daily listening.	Pre-test Post-test (16 weeks later)	Both groups, more in musical training: ↑ D-KEFS VF ↑ PASAT ↑ Stroop (Errors)	–
Seinfeld et al. [78]	2013	N = 29	60–85 years	24% men	Experimental (n = 13): piano training Active control (n = 16): leisure activities	No	4-month piano training. 1 and ½ h of group session and 45 min. of autonomous practice 5 days per week (~ 4 h).	4 months of non-musical leisure activities. Everyone chose at least one physical exercise course.	Pre-test Post-test (4 months later)	↑ FTT (D and ND) in both groups	↑ BDI in both ↑ POMS Total ↑ POMS Fatigue ↑ WHOQOL-BREF Physical ↑ WHOQOL-BREF Psychological
										In musical training: ↑ Stroop-Color ↑ Stroop-Color-Word ↑ DSF Trend in TMT-A	
Thorne [77]	2015	N = 20	65–85 years	35% men	Experimental (n = 10): piano training Active control (n = 10): musical listening	Yes	6-month piano training. ½ h of group session per week and ½ h of daily practice.	6-month musical listening. ½ h of group session per week and ½ h of daily listening.	Pre-test Post-test (6 months later) Follow-up (3 months later)	↑ Stroop (Errors) ↑ VBM delayed recall	↑ MOS Energy and Emotional Well-being ↑ MMN amplitude in experimental group after training: better auditory discrimination

**DSy**: Digit Symbol; **TMT**: Trail Making Test; **DSF**: Digit Span Forward; **D-KEFS**: Delis-Kaplan Executive Function System; **VF**: Verbal Fluency; **PASAT**: Paced Auditory Serial Addition Test; **FTT**: Finger Tapping Test; **VBM**: Verbal Memory; **BDI**: Beck Depression Inventory; **POMS**: Profile of Mood State; **WHOQOL-BREF**: The World Health Organization Quality of Life Brief Questionnaire; **MMN**: Mismatch Negativity.

In all the studies, a training program based on piano and musical language teaching was selected, with variations in the total program duration (between 4 and 6 months), type of lessons (3 studies with group lessons, and 1 study with individual lessons), duration of lessons (between 30 minutes and 1.5 hours per session) and autonomous practice time. Differences also existed in the type of control group used: passive control, music listening, or others with workshops involving various leisure activities (all the participants chose at least one physical activity). It should be noted that not all the studies carried out a random assignment of the participants [78]. A follow-up evaluation was conducted in two studies 3 months after the post-test. Lastly, as shown on Table 3, some diversity was also found in the neuropsychological tests used to assess cognitive functions.

#### Meta-analyses of specific cognitive functions

**Fig 7:**
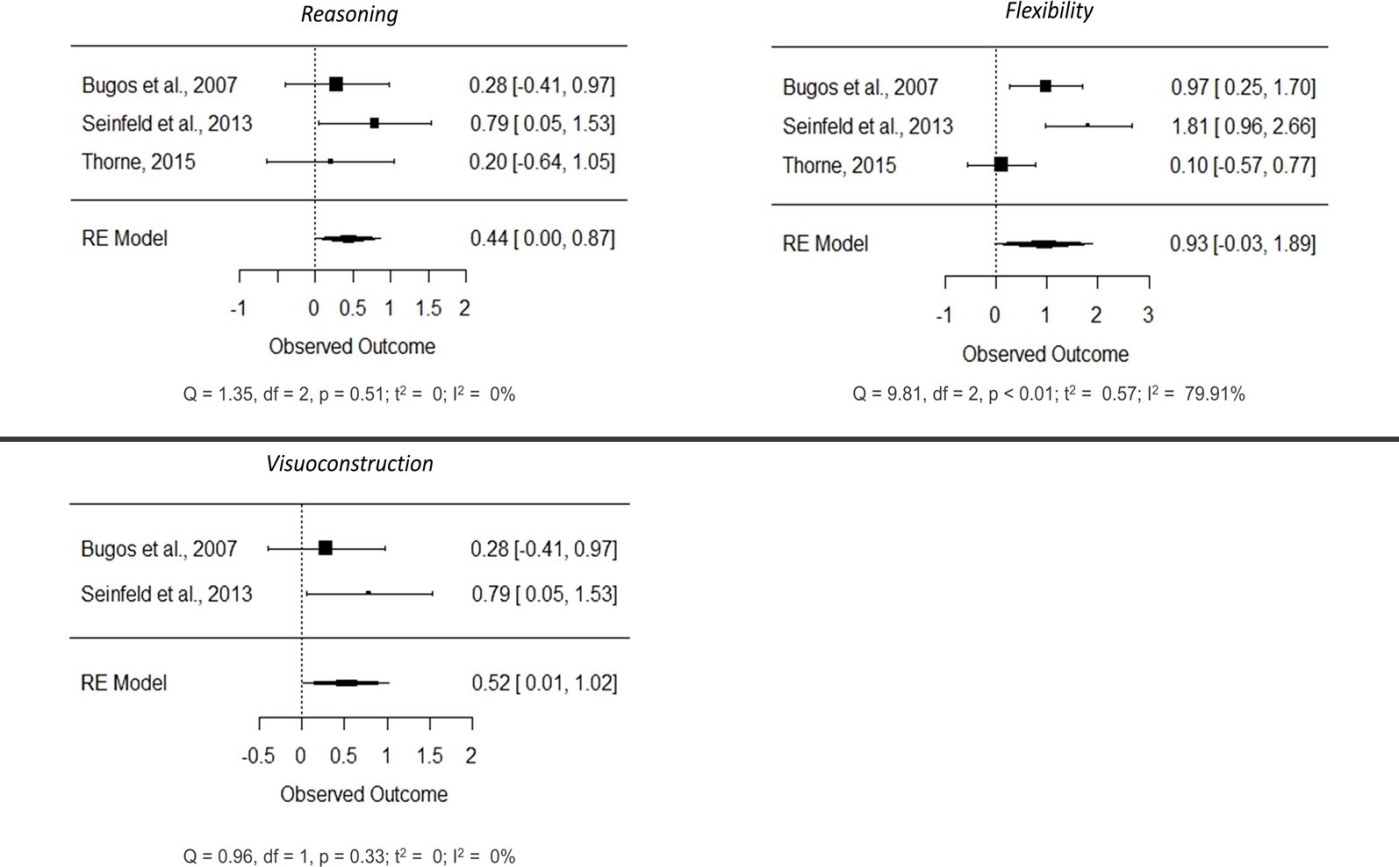
Forest plots showing the effects of short-term musical training on reasoning, visuoconstruction and flexibility in older adults.

As with the correlational studies, we conducted independent meta-analyses for each of the functions. Subsequently, we explored basic domain-general functions (processing speed, inhibition and attention) and complex domain-general functions (verbal working memory, verbal fluency, visuoconstruction, reasoning and flexibility), in addition to domain-specific functions (manual dexterity) (Fig 7; forest plots of non-significant cognitive functions are reported in the S3 File). These cognitive functions were not assessed in all of the studies, which caused the number of total samples for each of the meta-analyses to range between 49 and 126 participants (mean = 85, SD = 24,471). Overall, we found positive mean effect sizes in most functions (except verbal working memory), although only reasoning (SMD = 0.436, 95% CI[0.003, 0.869], p = 0.048; small effect) and visuoconstruction (SMD = 0.519, 95% CI[0.015, 1.024], p = 0.044; medium effect) reached statistical significance, and a certain trend was observed in flexibility (SMD = 0.934, 95% CI[-0.026, 1.894], p = 0.057; large effect). This suggests that musical training in older adults can lead to improvements in cognitive functions, especially in high-level functions such as reasoning, and probably in flexibility. Interestingly, verbal working memory exhibited a negative effect (SMD = -0.291, 95% CI [-1.318, 0.736], p = 0.579).

Most functions showed substantial heterogeneity between studies (I^2^ > 50%). However, this heterogeneity did not seem to be present in visuoconstruction and reasoning (p-values associated with the Q tests > 0.05; I^2^ values of 0% in both), which suggests that the variation observed across studies in the outcomes for this function can only be attributed to chance.

#### Other findings of the systematic review

In addition to the previous section, the systematic review provided further evidences. Along with the improvements observed in the meta-analyses, the only study in which long-term memory was explored [77] found a significant improvement in the delayed recall of verbal memory. EEG data also showed differences in pre-attentive processing [77]. In the aforementioned study, subjects who had received musical training showed a larger amplitude of the mismatch negativity (MMN) potential associated with sound discrimination. Interestingly, some of the benefits observed after training (in working memory [36] and in inhibition capacity [77]) were not maintained after this training ceased, as shown by subsequent follow-up assessments.

Moreover, some of the improvements observed in the meta-analyses did not consistently reach statistical significance in individual studies. It may be due to small sample sizes (and the consequent low statistical power) or the duration and intensity of the training programs. In fact, Bugos [79] was unable to replicate in 2010 either the significant improvements in divided attention, measured with the Trail Making Test B, or the trend in the B-A score found in the 2007 study by Bugos et al. [36]. In the 2010 study, both a reduction in sample size and a reduction in the number of months of musical training were observed (4 months in Bugos, 2010 vs. 6 months in Bugos et al., 2007).

## Discussion

The progressive aging of societies makes it necessary to identify ways to deal with the challenges of aging. In this context, musical practice seems to be a promising tool to reduce the impact of age-related cognitive and brain changes. Despite the increasing interest in music-based interventions nowadays, to our knowledge, this research is the first systematic review and meta-analysis of the available evidence on the effects of musical practice on healthy neurocognitive aging.

### Musical practice as a protective factor in neurocognitive aging

Overall, the evidence reviewed suggested a relationship between musical practice and better cognitive functioning in old age. The inclusion of studies with correlational and experimental designs revealed that these benefits were present both in individuals who had engaged in long-term musical practice throughout their lives (correlational studies) and in those who had followed short training programs initiated in later stages of life (experimental studies). These cognitive benefits, which were particularly noticeable in individuals who had engaged in long-term musical practice, were present in both domain-specific and domain-general functions. Specifically, older adults who practiced music showed improvements in the early processing of auditory stimuli; this was to be expected, given that musical training usually involves this type of processing. This had already been observed in studies with adults [39–44,80] and in other studies with older individuals, which could not be included in the current meta-analysis for methodological reasons (mainly due to the age criterion) [81–84]. Moreover, the benefits were also generalized to functions not so closely related to the specific skills trained by musical practice (i.e., domain-general functions), such as naming, episodic memory or executive functions. As happened with auditory processing, these improvements were consistent with research in adults (see Introduction) and other studies with older adults [81,84–87].

Importantly, most of the improvements observed occurred in age-sensitive cognitive functions, protecting or reducing the decline [74]. On the other hand, in functions that are less sensitive to the passage of time musical practice seems to be associated with an enhancing effect. Therefore, cognitive aging is not inevitable, and this variability seems to depend partly on lifelong experiences and lifestyle factors.

Moreover, it seems that some tests are more sensitive than others to cognitive differences between older musicians and older non-musicians. Specifically, an individual extreme effect was found in visuospatial ability in the Benton Visual Form Discrimination test (SMD = 5.044; [75]); in the same sample, another visuospatial ability test, the Judgment of Line Orientation test, only found a moderate effect (SMD = 0.508). In this case, a possible explanation could be that the Benton Visual Form Discrimination test requires making fine discriminations between very similar shapes, a skill that professional musicians may train by reading scores with similar-looking stimuli. However, the orientation of these musical symbols does not change, since what is really informative about them is their position (height) on the musical staff. A possibility yet to be confirmed is whether certain position discrimination tests will also show a high sensitivity to the effects of musical practice.

Likewise, larger effects were found in the most demanding tasks within the same function, as happened in working memory (Listening Span Test and Visual Pattern Test Active [37]), naming (with a 30-item version of the Boston Naming Test [64]), verbal fluency (with 2-minute versions instead of the usual 1-minute version [74]), and verbal memory (in the long version of the California Verbal Learning Test-II compared to the short version [75]). This is consistent with the *complexity effect* [11]: just like an increase in the demands of the task is likely to affect older people more than younger people, older musicians may be less influenced by the demands due to their improved cognitive functioning.

As could be expected, the improvements were much more evident in the correlational studies, given that such studies explored older adults with an extensive musical practice and an early onset. However, the correlational evidence cannot be used to identify the directionality of the relationship between musical practice and cognitive functioning. Alternatively, the specific cognitive characteristics of older musicians may have led them to select and maintain this activity throughout their lives (as a predispositional factor); therefore, the fact of choosing groups of musicians may imply selecting a sample with a particular cognitive profile.

However, all the studies attempted to reduce this limitation by controlling for other variables (e.g., education or crystallized cognition). In addition, it appears that variables related to musical experience were also associated with the observed cognitive effects. Although these are correlational findings, the fact that the characteristics of musical practice have a relationship with cognitive performance again supports the idea of possible causality. Among these musical variables, the relationship between the onset of musical practice and age suggests the possibility of a sensitive period during which a long-term enhancement takes place and can be observed even in late stages of life. Specifically, White-Schwoch et al. [88] observed that the neurocognitive effects of early musical practice in older adults were permanent, as they could be observed even when this practice had been discontinued after 25 years.

Nevertheless, the best way to resolve the issue of causal directionality is to use an experimental methodology. Even though experimental studies were few and involved relatively short training programs, the meta-analyses of these works showed an overall positive trend in most of the functions explored, along with clear effects in reasoning and visuoconstruction (significant) and flexibility (marginally significant). This supports the idea that plasticity processes are maintained in the brain of adult and aged individuals [33,34] and that musical activity may continue to stimulate such changes beyond childhood (even with short-term experiences). However, the continuity of these effects is not yet clear, given that some improvements were not maintained in the follow-up assessments.

By contrast, a surprising result was the negative effect observed in verbal working memory in the experimental studies. However, it seems that this was due to the influence of the effect in Seinfeld et al. [78], in which the groups were not equated in the baseline and a highly active control was chosen (i.e., weekly physical exercise workshops, among other activities).

### Explanations on the cognitive benefits of musical practice

Although the causal role of musical practice cannot be established yet, a number of findings indicated that some of the differences observed were due to this lifestyle factor. As previously mentioned, musical performance triggers a large number of cognitive processes, which may have a specific impact on each one of these processes by reinforcing their function (*specific training mechanism*). Specifically, musical performance continuously involves both musical listening and sound discrimination (auditory processing) [79], as well as the rapid reading of musical scores and the analysis of the location of notes on the staff (linguistic and visuospatial ability) [86]. This activity also involves many attentional demands: playing in a group requires synchronizing one’s own performance (sensorimotor skills) and listening to the musical cues of other instruments (audio-spatial localization) [86], attending to many types of visual stimuli, mainly the score and the body movements of the rest of the musicians and the conductor (divided attention), detecting them and responding appropriately (vigilance and selective attention) over long periods of time (sustained attention) [47]. Regarding the executive functions, musicians must inhibit other information while performing (e.g., other melodies) [37] and must apply monitoring and shifting skills during the performance [89]. Lastly, in many cases musical performance requires the ability to memorize the musical piece and recall it at the time of the concert [71].

However, the improvements observed in our results in older musicians are very broad and cover a wider range of cognitive functions. The findings suggest the existence of a transfer effect of musical practice, which is particularly relevant given the difficulties that many cognitive training programs have obtaining far transfer [36]. In fact, the only cognitive benefits that achieved statistical significance in the meta-analyses of the experimental studies were found in domain-general functions such as visuoconstruction, reasoning and flexibility.

Therefore, improvements in certain functions could have a positive influence on other non-trained processes (*specific compensatory mechanism*). First, it has been noted that improvements in auditory processing are not limited to musical tones but also extend to speech processing. These improvements are likely to affect high-level functions within the verbal context, such as language comprehension in noisy contexts (speech-in-noise; [51,85]). Such linguistic benefits seem to be related to the existence of common brain processing mechanisms for music and language, as proposed in Patel’s OPERA hypothesis [90]. Consequently, improvements in one of these two functions can be expected to have a transfer effect on the other function [91]. However, that influence could be specific to some processes instead of general (auditory improvements were not associated with the observed improvements in working memory and visuospatial tests [37]).

Another example can be found in the case of long-term memory. Additionally to the contribution of a higher perceptive ability [92], further evidence suggests that the benefits of musical practice on memory are not due *per se* to improved storage or retrieval capacity, but rather to a more robust and efficient coding (such as an improved rehearsal mechanism [93] or an increased use of semantic information organization strategies [94]).

Finally, an explanation for our results could be drawn from certain aging theories. Just like the decline in certain key functions during aging mediates changes in many other cognitive processes, an improvement or preservation of these key functions may lead to an improved overall functioning (*general compensatory mechanism*). Thus, the presence in musicians of a more efficient processing [8,9] along with a higher inhibitory capacity [10] could be at the root of the other benefits observed. A similar approach has been proposed previously with inhibitory control for adult enhancements [95]. In our opinion, based on the evidence found, attention is also a function that has a crosscutting influence on the rest of the cognitive processes. Attention has been found to be able to contribute to benefits in auditory processing [44,83,96], visuospatial ability [49] and working memory [80] in musicians, among other functions. However, none of the three mechanisms described is exclusive of the others, and they may all occur simultaneously.

### Limitations and implications

This review highlights the limited research conducted in this field, even though it is a promising subject. However, the results of our meta-analyses should be interpreted with caution due to the small number of studies included and the amount of heterogeneity observed. Some of this heterogeneity is likely to be due to differences in the tasks used, educational level and age of the samples, differences in musical variables (in correlational studies), and differences in the duration and intensity of scheduled practice, as well as the type of control group used (in the experimental studies). Another difference between the correlational studies was that musicians in some cases were professionals and in other amateurs (although the majority were instrumentalists).

The limitations of our study imply that the evidence provided cannot be considered conclusive. However, the results obtained are positive and consistent with the adult literature. It is therefore essential to conduct new research. Especially, there is a need for further experimental studies involving training programs with a random assignment of the participants, since it is the only way to rule out that the differences found in musicians are due to predispositional factors. In addition, it would be interesting to explore the differential effect on aging in professional versus amateur practice, and in the various types of musical practice (orchestral musicians, jazz, flamenco, singers, composers, conductors, etc.) due to the idiosyncratic cognitive demands that each of them implies. There is also a lack of neuroimaging studies. To our knowledge, the only one conducted [86] showed that musical practice could also have a protective effects on the brain.

Moreover, our results have strong implications in the contexts of education and society. The possibility that early musical practice may produce long-term neurocognitive benefits could lead to rethinking the subject of music in the school context. As shown by this review, intense musical activities during childhood could increase the probability of healthier neurocognitive aging and reduce the risk of neurodegenerative diseases. In addition, musical practice during later stages of life could also be an effective factor to take into account in the design of intervention programs for the prevention of age-related neurocognitive problems.

## Conclusions

Age-related cognitive decline does not seem to be inevitable, given that lifestyle factors such as musical practice have been associated with improved cognitive functioning during aging. In this paper, a systematic review and meta-analysis of the impact of musical practice on neurocognitive aging were conducted. Results indicate that an involvement in this activity (particularly early and long-term involvement) is associated with benefits in domain-specific functions (auditory perception) and in a wide range of domain-general functions. Although little evidence is available so far and further research is needed, the findings presented here suggest that musical practice is an effective tool for preventing the declines of healthy aging and making interventions in this regard.

## Supporting information

S1 Table PRISMA checklist. (DOCX)

S1 File Search procedures of each database. (DOCX)

S2 File Coding sheets for the recording of variables in the studies included in the meta-analysis. (DOCX)

S2 Table Cognitive functions explored with independent meta-analyses, including aggregates produced in studies where multiple outcomes existed for the same function. (DOCX)

S3 File Forest plots of non-significant cognitive functions in correlational studies. (DOCX)
