# Supplementary material for: Musical practice as an enhancer of cognitive function in healthy aging - A systematic review and meta-analysis
Source: PLoS One. 2018 Nov 27;13(11):e0207957. doi: 10.1371/journal.pone.0207957 (PMC6258526; doi:10.1371/journal.pone.0207957)
Supplement: S1 File — (DOCX) [file pone.0207957.s002.docx]

**SEARCH PROCEDURES**

On August 2018 the first author (RRC) conducted an electronic search on the Ovid, Proquest, Pubmed and Scopus databases, as well as the Web of Science platform. He entered the search equation *(aging OR older* OR elder*) AND (music* OR musical practice OR musical training) AND (cogniti* OR cognitive reserve OR plasticit*)* into the Title field (in all of them), and Abstract field (Ovid, ProQuest, PubMed and Scopus) or Subject field (Web of Science). The search was specifically limited in each case:

**Ovid**

- No restrictions

**ProQuest**

1. Articles in English or Spanish
2. Type of documents restricted to “article”, “main article” and “dissertation/thesis”

**PubMed**

- No restrictions

**Scopus**

1. Articles in English or Spanish
2. Type of documents restricted to “article” and “article in press”
3. Subject areas restricted to “psychology”, “neurosciences” and “medicine”

**Web of Science**

1. Articles in English or Spanish
2. Type of documents restricted to “article”
3. Research areas restricted to “psychology”, “behavioral sciences”, “neurosciences/ neurology” and “geriatrics/gerontology”

This initial search allowed the identification of 405 potentially interesting studies in Ovid, 738 in ProQuest, 2 in PubMed, 10 in Scopus and 543 in Web of Science. Subsequently, 855 duplicates were removed, and titles and abstracts of 843 studies were screened by the first author to exclude articles that did not meet the inclusion criteria (C1-C6). This resulted in 37 full-text articles assessed for eligibility by two independent reviewers (RRC and JL). Any disagreements were resolved by discussion and consensus between both researchers until 100% agreement was reached. Finally, 13 of these articles were selected for inclusion in the review.
