# Supplementary material for: Musical practice as an enhancer of cognitive function in healthy aging - A systematic review and meta-analysis
Source: PLoS One. 2018 Nov 27;13(11):e0207957. doi: 10.1371/journal.pone.0207957 (PMC6258526; doi:10.1371/journal.pone.0207957)
Supplement: S2 File — (DOCX) [file pone.0207957.s003.docx]

## CODING SHEETS

**1. CORRELATIONAL STUDIES**

**General coding sheet for correlational studies**

| Nº | Variable | Levels |
| --- | --- | --- |
| 1 | Authors |  |
| 2 | Year of publication |  |
| 3 | Country |  |
| 4 | Language |  |
| 5 | Main purpose |  |
| SUBSTANTIVE ASPECTS | | |
| 6 | Age of the sample (mean and standard deviation) Edad (Media y DT) de la muestra |  |
| 7 | Sex of the sample (% men) |  |
| METHODOLOGICAL ASPECTS | | |
| 8 | Type of design (cross-secgtional or longitudinal) |  |
| 9 | Total sample size |  |
| 10 | Group type (characteristic) and size |  |
| 11 | Moments of measures (pretest, postest, follow-up) |  |
| 12 | Blind evaluation (yes or no) |  |
| 13 | Tests or evaluation techniques |  |
| 14 | Evaluated cognitive variables |  |
| 15.1 | Age of onset (mean and standard deviation) |  |
| 15.2 | Years of formal musical education (mean and standard deviation) |  |
| 15.3 | Years of practice mean and standard deviation) |  |
| 15.4 | Musical aptitude (instrument, mean and standard deviation) |  |
| 15.5 | Current practice (% of participants) |  |
| 16 | Main results |  |

- **Observations**:

**Coding sheet for the most relevant control variables**

| Nº | Variable | Levels |
| --- | --- | --- |
| I | Years of education (mean and standard deviation) |  |
| II | Lifelong physical activity |  |
| III | Current physical activity (% of participants) |  |
| IV | Intelligence (instrument, mean and standard deviation) |  |
| V | Depression |  |
| VI | Absense of psychiatric and neurological deseases |  |
| VII | Absense of alcoholism, drug abuse and psychoactive medication |  |

- **Others (evaluation method, mean and standard deviation)**:

**2. EXPERIMENTAL STUDIES**

**General coding sheet for experimental studies**

| Nº | Variable | Levels |
| --- | --- | --- |
| 1 | Authors |  |
| 2 | Years of publication |  |
| 3 | Country |  |
| 4 | Language |  |
| 5 | Main purpose |  |
| SUBSTANTIVE ASPECTS | | |
| 6 | Age of the sample (mean and standard deviation) Edad (Media y DT) de la muestra |  |
| 7 | Sex of the sample (% men) |  |
| METHODOLOGICAL ASPECTS | | |
| 8 | Type of design (cross-secgtional or longitudinal) |  |
| 9 | Total sample size |  |
| 10 | Group type (characteristic) and size |  |
| 11.1 | Random assigment |  |
| 11.2 | Intervention program |  |
| 11.3 | Duration of the program |  |
| 12 | Moments of measures (pretest, postest, follow-up) |  |
| 13 | Blind evaluation (yes or no) |  |
| 14 | Tests or evaluation techniques |  |
| 15 | Evaluated cognitive variables |  |
| 16 | Main results |  |

- **Observations**:

**Coding sheet for the most relevant control variables**

| Nº | Variable | Levels |
| --- | --- | --- |
| I | Years of education (mean and standard deviation) |  |
| II | Lifelong physical activity |  |
| III | Current physical activity (% of participants) |  |
| IV | Intelligence (instrument, mean and standard deviation) |  |
| V | Depression |  |
| VI | Absense of psychiatric and neurological deseases |  |
| VII | Absense of alcoholism, drug abuse and psychoactive medication |  |

- **Others (evaluation method, mean and standard deviation)**:
