# Supplementary material for: Musical practice as an enhancer of cognitive function in healthy aging - A systematic review and meta-analysis
Source: PLoS One. 2018 Nov 27;13(11):e0207957. doi: 10.1371/journal.pone.0207957 (PMC6258526; doi:10.1371/journal.pone.0207957)
Supplement: S2 Table — (DOCX) [file pone.0207957.s004.docx]

**NEUROPSYCHOLOGICAL AGGREGATES**

**Correlational Studies**

| AGGREGATE | STUDY | OUTCOMES |
| --- | --- | --- |
| Processing Speed | Fauvel et al., 2014 | - Digit-symbol coding of the WAIS-III - d2 test |
|  | Hanna-Pladdy & Gajewski, 2012 | - D-KEFS Trails 1 - Finger Tapping Test-right hand - Finger Tapping Test-left hand |
|  | Hanna-Pladdy & MacKay, 2011 | - Trail Making Test A |
|  | Mansens, Deeg & Comijs, 2017 | - Alphabet Coding Task-15 |
|  | Moussard et al., 2016 | - Go RT |
|  | Strong & Midden, 2018 | - Trail Making Test A |
| Verbal Memory | Baird et al., 2017 | - Total learning - Trials to criterion - 30-min recall - 24-hour recall - Recognition accuracy |
|  | Fauvel et al., 2014 | - Delayed recall of the Signoret BEM-144’S 12 words |
|  | Hanna-Pladdy & Gajewski, 2012 | - California Verbal Learning Test-II, total learning - CVLT-II, short delay free recall - CVLT-II, long delay free recall |
|  | Hanna-Pladdy & MacKay, 2011 | - California Verbal Learning Test-II, total learning - CVLT-II, short delay free recall - CVLT-II, long delay free recall |
|  | Mansens, Deeg & Comijs, 2017 | - Auditory Verbal Learning Test, learning - AVLT, delayed recall - AVLT, retention |
|  | Strong & Midden, 2018 | - California Verbal Learning Test-II, total learning - CVLT-II, long delay free recall |
| Visual Memory | Fauvel et al., 2014 | - Door test - Rey-Osterrieth Complex Figure, delayed recall |
|  | Hanna-Pladdy & Gajewski, 2012 | - Visual Reproduction-I (immediate recall) of WMS-III - Visual Reproduction-II (delayed recall) of WMS-III - Rey-Osterrieth Complex Figure, immediate recall - Rey-Osterrieth Complex Figure, delayed recall |
|  | Hanna-Pladdy & MacKay, 2011 | - Visual Reproduction-I (immediate recall) of WMS-III - Visual Reproduction-II (delayed recall) of WMS-III |
|  | Strong & Midden, 2018 | - Brief Visuospatial Memory Test-R, Total immediate - BVM-R, delayed recall |
| Verbal Fluency | Fauvel et al., 2014 | - Phonemic fluency - Semantic fluency |
|  | Hanna-Pladdy & Gajewski, 2012 | - Letter fluency - Semantic fluency |
|  | Hanna-Pladdy & MacKay, 2011 | - Letter fluency - Semantic fluency |
|  | Mansens, Deeg & Comijs, 2017 | - Letter fluency - Semantic fluency |
|  | Strong & Midden, 2018 | - Letter fluency - Semantic fluency |
| Phonological fluency | Fauvel et al., 2014 | - Phonemic fluency |
|  | Hanna-Pladdy & Gajewski, 2012 | - Letter fluency |
|  | Hanna-Pladdy & MacKay, 2011 | - Letter fluency |
|  | Mansens, Deeg & Comijs, 2017 | - Letter fluency |
|  | Strong & Midden, 2018 | - Letter fluency |
| Semantic fluency | Fauvel et al., 2014 | - Semantic fluency |
|  | Hanna-Pladdy & Gajewski, 2012 | - Semantic fluency |
|  | Hanna-Pladdy & MacKay, 2011 | - Semantic fluency |
|  | Mansens, Deeg & Comijs, 2017 | - Semantic fluency |
|  | Strong & Midden, 2018 | - Semantic fluency |
| Naming | Hanna-Pladdy & Gajewski, 2012 | - Boston Naming Test |
|  | Hanna-Pladdy & MacKay, 2011 | - Boston Naming Test |
|  | Strong & Midden, 2018 | - Boston Naming Test |
| Reasoning | Fauvel et al., 2014 | - Raven’s progressive matrices test |
|  | Hanna-Pladdy & Gajewski, 2012 | - Wisconsin Card Sorting Task-categories - Tower-total |
|  | Strong & Midden, 2018 | - Block Design of WAIS-III |
| Inhibition | Grassi et al., 2017 | - Listening Span Test-Intrusion |
|  | Moussard et al., 2016 | - No-go correct (%) |
|  | Strong & Midden, 2018 | - D-KEFS Stroop Color-Word 3 |
| Flexibility | Hanna-Pladdy & Gajewski, 2012 | - D-KEFS switching fluency - Wisconsin Card Sorting Task-perseverations - Wisconsin Card Sorting Task-categories |
|  | Hanna-Pladdy & MacKay, 2011 | - Trail Making Test B |
|  | Strong & Midden, 2018 | - Trail Making Test difference B-A - D-KEFS Stroop Color-Word 4 |
| Verbal Working Memory | Fauvel et al., 2014 | - Forward digit span |
|  | Grassi et al., 2017 | - Listening Span Test |
|  | Hanna-Pladdy & Gajewski, 2012 | - Digit span of WAIS-III - Letter-Number Sequencing of WAIS-III |
|  | Hanna-Pladdy & MacKay, 2011 | - Digit span of WAIS-III - Letter-Number Sequencing of WAIS-III |
|  | Mansens, Deeg & Comijs, 2017 | - Digit span forward - Digit span backward |
|  | Strong & Midden, 2018 | - Digit span of WAIS-IV |
| Visual Working Memory | Grassi et al., 2017 | - Corsi Forward - Corsi Backward - Visual Pattern Test Active |
|  | Hanna-Pladdy & Gajewski, 2012 | - Spatial span of WMS-III |
|  | Hanna-Pladdy & MacKay, 2011 | - Spatial span of WMS-III |
|  |  |  |
| Manual Dexterity | Hanna-Pladdy & Gajewski, 2012 | - Grooved Pegboard Test-right hand - Grooved Pegboard Test-left hand - Finger Tapping Test-right hand - Finger Tapping Test-left hand |
| Visuospatial ability | Grassi et al., 2017 | - Short Embedded Figures Test - Short Mental Rotation Test |
|  | Hanna-Pladdy & Gajewski, 2012 | - Benton Judgment of Line Orientation - Benton Visual Form Discrimination |
|  | Strong & Midden, 2018 | - Benton Judgment of Line Orientation |
| Visuoconstruction | Hanna-Pladdy & Gajewski, 2012 | - Rey-Osterrieth Complex Figure, copy |
|  | Strong & Midden, 2018 | - Block Design of WAIS-III |
| Attention | Fauvel et al., 2014 | - d2 test |
|  | Hanna-Pladdy & Gajewski, 2012 | - D-KEFS Trails 4 |
|  | Hanna-Pladdy & MacKay, 2011 | - Trail Making Test A - Trail Making Test B |
|  | Strong & Midden, 2018 | - Trail Making Test A - Trail Making Test B - D-KEFS Stroop Color-Word 3 |

**Experimental Studies**

| AGGREGATE | STUDY | OUTCOMES |
| --- | --- | --- |
| Processing Speed | Bugos et al., 2007 | - Digit Symbol of WAIS-III - Trail Making Test A |
|  | Bugos, 2010 | - Paced Serial Addition Task - Trail Making Test A |
|  | Seinfeld et al., 2013 | - Finger Tapping Test-right hand - Finger Tapping Test-left hand - Trail Making Test A - Symbol Digit Modalities Test |
|  | Thorne, 2011 | - Finger Tapping Test-right hand - Finger Tapping Test-left hand - Symbol Digit Coding-correct - Symbol Digit Coding-correct RT |
| Attention | Bugos et al., 2007 | - Trail Making Test A - Trail Making Test B |
|  | Bugos, 2010 | - Paced Serial Addition Task - Trail Making Test A - Cued Color-Word Stroop Test |
|  | Seinfeld et al., 2013 | - Trail Making Test A - Trail Making Test B - Stroop Color-Word |
|  | Thorne, 2011 | - Stroop Color-Word-correct RT - Continuous Performance Test-correct - Trail Making Test A - Trail Making Test B |
| Manual Dexterity | Seinfeld et al., 2013 | - Grooved Pegboard Test-right hand - Grooved Pegboard Test-left hand - Finger Tapping Test-right hand - Finger Tapping Test-left hand |
|  | Thorne, 2011 | - Finger Tapping Test-right hand - Finger Tapping Test-left hand |
| Verbal Working Memory | Bugos et al., 2007 | - Digit Span of WAIS-III - Letter-Number Sequencing of WAIS-III |
|  | Bugos, 2010 | - Paced Serial Addition Task |
|  | Seinfeld et al., 2013 | - Digit Span of WAIS-III-Forward - Digit Span of WAIS-III-Forward Amplitude - Digit Span of WAIS-III-Backward - Digit Span of WAIS-III-Backward Amplitude |
| Verbal Fluency | Bugos, 2010 | - D-KEFS Verbal Fluency (total) |
|  | Seinfeld et al., 2013 | - Letter Fluency |
| Reasoning | Bugos et al., 2007 | - Block Design of WAIS-III |
|  | Seinfeld et al., 2013 | - Block Design of WAIS-III |
|  | Thorne, 2011 | - Non Verbal Reasoning Test-correct |
| Flexibility | Bugos et al., 2007 | - Trail Making Test B |
|  | Seinfeld et al., 2013 | - Trail Making Test B |
|  | Thorne, 2015 | - Trail Making Test B - Shifting Attention-correct - Shifting Attention-errors - Shifting Attention-correct RT |
| Inhibition | Bugos, 2010 | - Cued Color-Word Stroop Test |
|  | Seinfeld et al., 2013 | - Stroop Color-Word |
|  | Thorne, 2011 | - Stroop Color-Word-errors - Continuous Performance Test-errors |
| Visuoconstruction | Bugos et al., 2007 | - Block Design of WAIS-III |
|  | Seinfeld et al., 2013 | - Block Design of WAIS-III |
