# Supplementary material for: Musical practice as an enhancer of cognitive function in healthy aging - A systematic review and meta-analysis
Source: PLoS One. 2018 Nov 27;13(11):e0207957. doi: 10.1371/journal.pone.0207957 (PMC6258526; doi:10.1371/journal.pone.0207957)
Supplement: S3 File — (DOCX) [file pone.0207957.s005.docx]

**NON-SIGNIFICANT COGNITIVE FUNCTIONS IN CORRELATIONAL STUDIES**


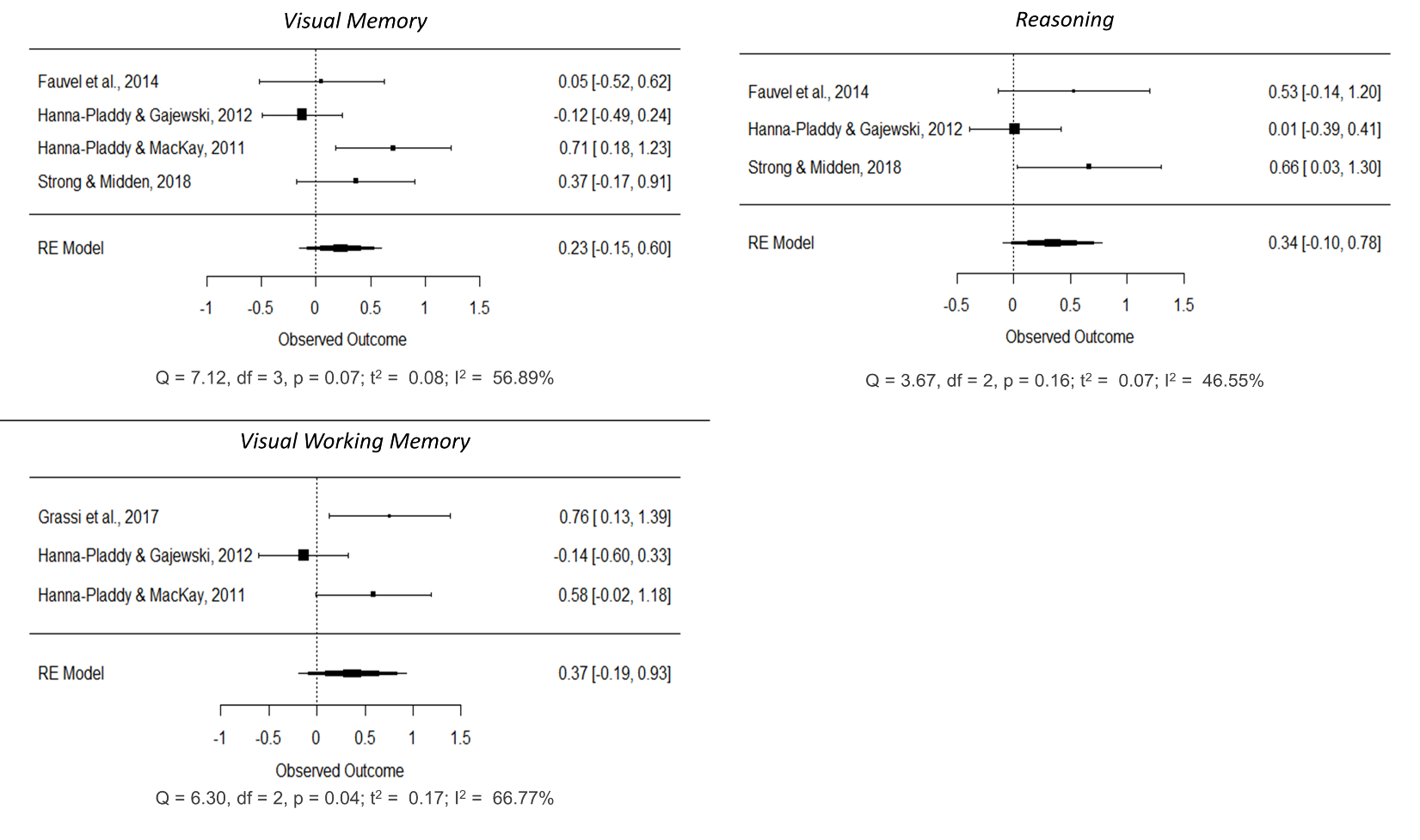


**Forest plots of the effect of longlife musical practice on visual memory, visual working memory and reasoning during aging.**

**NON-SIGNIFICANT COGNITIVE FUNCTIONS IN EXPERIMENTAL STUDIES**

**
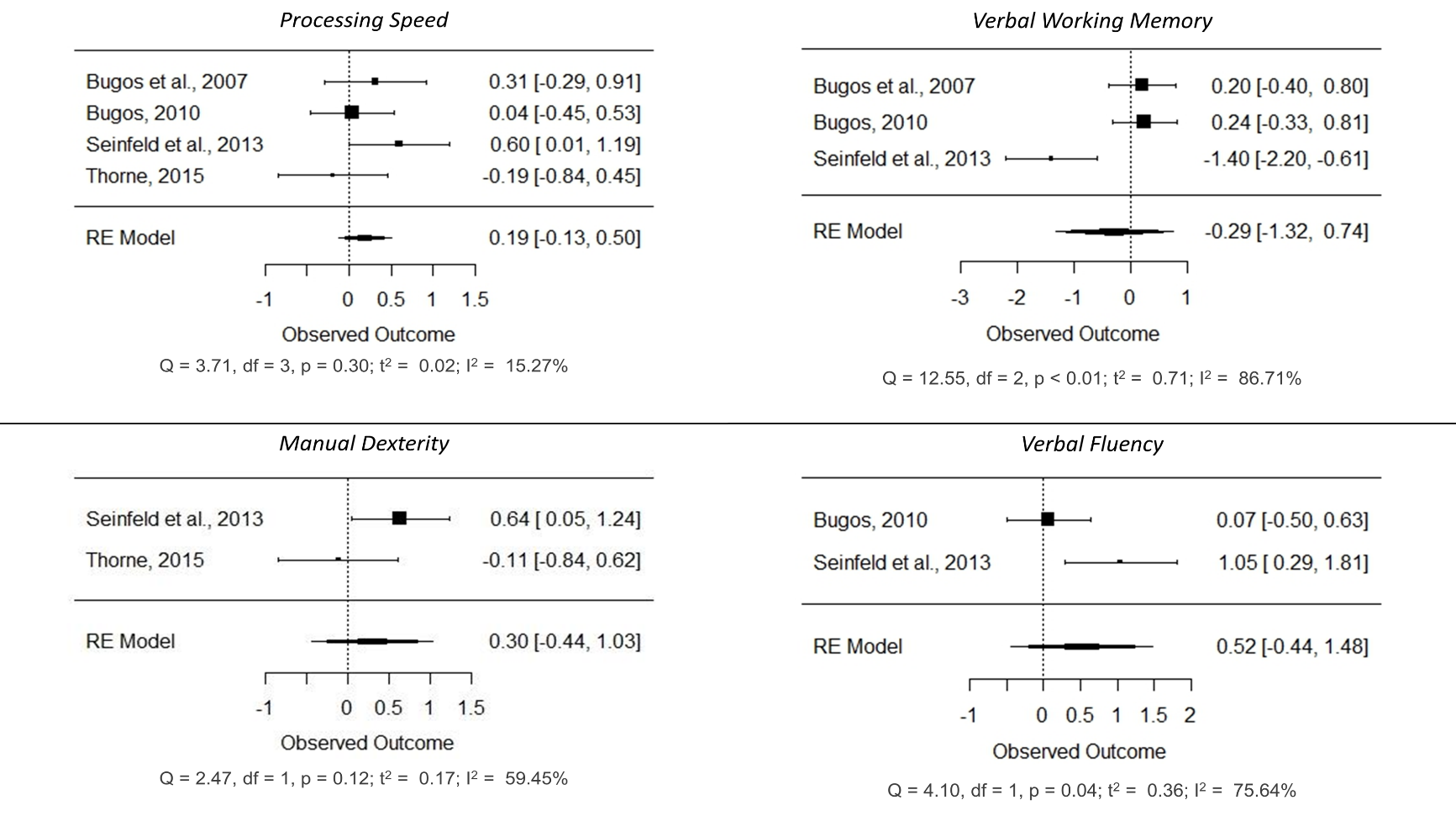
**

**Forest plots of the effect of short-term musical training on processing speed, manual dexterity, verbal working memory and verbal fluency during aging.**

**
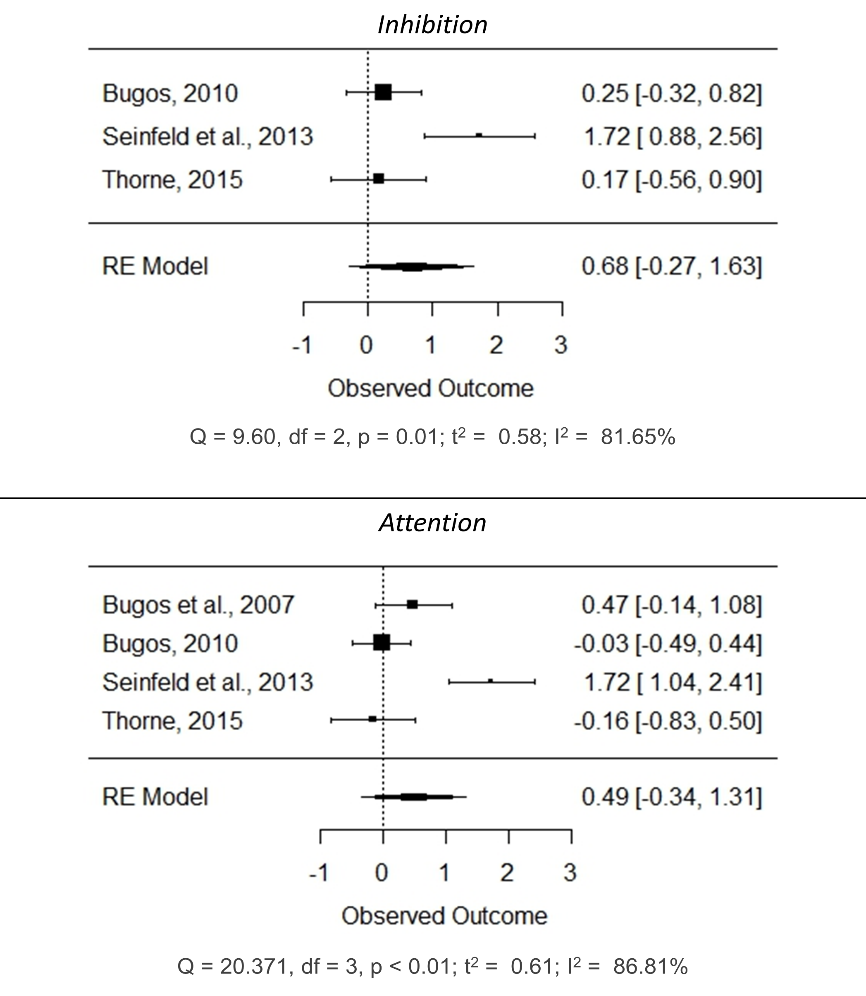
**

**Forest plots of the effect of short-term musical training on inhibition and attention during aging.**
